# Impact of massive hemorrhage on outcome in patients with orthotopic liver transplant: A retrospective unicenter study

**DOI:** 10.1097/MD.0000000000043575

**Published:** 2025-07-25

**Authors:** Iulian Buzincu, Mihaela Blaj, Adi-Ionut Ciumanghel, Irina Gîrleanu, Eliza Isabela Bărbuță, Irina Ciumanghel, Ana-Maria Trofin, Cristian Dumitru Lupașcu

**Affiliations:** a “Grigore T. Popa’’ University of Medicine and Pharmacy, Iasi, Romania; b Anesthesia and Intensive Care, “St. Spiridon” Emergency Clinical County Hospital, Iasi, Romania; c Institute of Gastroenterology and Hepatology, “St. Spiridon” Emergency Clinical County Hospital, Iasi, Romania; d Emergency Department, “St. Spiridon” Emergency Clinical County Hospital, Iasi, Romania; e General Surgery and Liver Transplant Clinic, “St. Spiridon” Emergency Clinical County Hospital, Iasi, Romania.

**Keywords:** liver transplant, massive hemorrhage, medical complications, mortality, surgical complications

## Abstract

Despite advancements in surgical and anesthetic techniques, significant bleeding remains a common challenge during orthotopic liver transplantation (OLT). The extensive nature of the surgical procedure, combined with the patient’s fragile coagulation profile, places individuals undergoing OLT at a high risk for massive hemorrhage (MH). Blood transfusions and hemostatic products are frequently required and can be life-saving. However, these resources are often limited, and their use may be associated with numerous complications. This study aimed to evaluate the impact of MH on the day of surgery on the outcomes of patients undergoing OLT from brain-dead donors at our institution. We conducted a retrospective single-center study including all adult patients who underwent OLT from brain-dead donors at the Emergency County Clinical Hospital “Saint Spiridon” in Iași. Preoperative circulating blood volume was calculated for each patient and compared with the estimated blood loss on the day of surgery. Patients who lost >1 circulating blood volume were classified as having experienced MH. The study population was divided into 2 groups: those with MH and those without. Outcomes were assessed based on 30-day and 1-year mortality, as well as medical and surgical complications. The study included 53 patients, with a median blood loss of 5500 mL (4650 mL). MH occurred in 26 patients (49.1%). Patients with a lower body mass index (24.3 vs 26.6, *P* = .028) and thrombocytopenia (55.5 vs 75 × 10³/µL, *P* = .04) were more likely to experience MH. Those with MH had higher rates of cardiovascular complications (38.5% vs 7.4%, *P* = .007), infectious complications (69.2% vs 37%, *P* = .009), and surgical reintervention (34.6% vs 11.1%, *P* = .04). Mortality rates at 30 days and 1 year did not significantly differ between patients with and without MH. MH adversely affects the outcomes of OLT patients, as evidenced by increased rates of cardiovascular and infectious complications and a higher likelihood of requiring surgical reintervention.

## 1. Introduction

Liver transplantation is the only definitive and life-saving treatment for patients with end-stage liver disease or hepatocellular carcinoma.^[1]^ Despite being a highly complex procedure associated with significant morbidity and mortality,^[2]^ advancements in patient selection, surgical and anesthetic techniques, perioperative care, and immunosuppressive therapies have significantly improved patient outcomes in recent years.^[3]^ However, massive intraoperative bleeding and challenging hemostasis due to cirrhotic coagulopathy remain major contributors to postoperative complications and mortality in liver transplantation.^[4]^

Patients with liver cirrhosis awaiting transplantation exhibit a fragile coagulation balance.^[5]^ Liver cirrhosis is often characterized by simultaneous reductions in both procoagulant and anticoagulant factors, predisposing these patients to both hemorrhagic and thrombotic complications.^[6]^ This coagulopathy is highly complex, involving dysfunctions in primary and secondary hemostasis and the fibrinolytic system.^[7]^ Key features include decreased fibrinogen levels, thrombocytopenia, and reduced levels of most coagulation factors, with the exception of factor VIII.^[8]^ Notably, platelet adhesiveness and aggregation are preserved despite thrombocytopenia.^[9]^ While procoagulant factor levels are diminished, thrombin generation in the presence of thrombomodulin remains normal.^[10]^ Although thrombotic events can occur, hemorrhagic complications are more common, particularly in the context of invasive procedures, including surgery.^[11]^

Beyond coagulopathy, cirrhotic patients also exhibit significant cardiovascular, pulmonary, and renal dysfunctions, further elevating their risk of intraoperative bleeding.^[12]^ Therefore, the surgical intervention is often accompanied by massive hemorrhage (MH), an aspect that worsens the outcome of this type of already fragile patient for whom intraoperative blood loss, blood transfusion and surgical reintervention are the main prognostic factor of mortality and morbidity.^[13]^

Managing bleeding in this setting is a significant challenge for anesthesiologists.^[14]^ Although coagulation management guided by viscoelastic tests has reduced the unnecessary use of natural and synthetic blood products,^[15]^ patients often still require large amounts of blood transfusions or the administration of hemostatic products.

While blood transfusions are critical, life-saving interventions, they are not without risks. Limited availability of blood products in hospital settings underscores the importance of their judicious use. Adverse effects of transfusions include allergic and hemolytic reactions, febrile nonhemolytic reactions, sepsis, non-cardiogenic pulmonary edema (transfusion-related acute lung injury), and circulatory overload (transfusion-associated circulatory overload). Furthermore, transfusions can paradoxically exacerbate bleeding through hemodynamic changes.^[16,17]^

This study aims to assess the impact of MH on the day of surgery on the outcomes of patients undergoing orthotopic liver transplant (OLT) from brain-dead donors at a tertiary care center, the Emergency County Clinical Hospital “Saint Spiridon” in Iași.

## 2. Materials and methods

### 2.1. Study patients

In this retrospective observational study, we included all adult patients with OLT from brain-dead donor performed in our tertiary center, between June 2016 to August 2024. We evaluated in-hospital postoperative complications. After discharge, all the patients were follow-up for 1 year. Mortality was evaluated at 30 days and 1 year.

### 2.2. Study design

The aim of the study was to evaluate the impact of MH on the day of surgery on the outcome in patients with OLT from a brain-dead donor. The study was performed in the surgery and in the intensive care unit departments of an university hospital, the largest in the region of Moldova, Romania, which has a total of 1153 beds.

The study group was divided into 2 subgroups according to the presence or absence of MH. We considered patients with MH those who lost >1 circulating blood volume (CBV) on the day of surgery.^[18]^ CBV was calculated using the formula: CBV = 70 mL × weight (kg), for all patients.

The coagulopathy was evaluated using standard coagulation tests (INR, aPTT, fibrinogen, platelets) and viscoelastic tests (trombelastometry).

The study was conducted in accordance with the principles outlined in the Declaration of Helsinki and received approval from the Hospital Ethics Committee No. 102 on November 14, 2024.

### 2.3. The anesthetic guideline for liver transplantation used in our hospital

Patients compatible in the AB0 system are hospitalized the day before the liver transplant in the Gastroenterology department of the same hospital. They are preoperatively multidisciplinary investigated, and if no absolute contraindication is detected, the patient with a higher disease severity is chosen, usually according to the Model of End-Stage Liver Disease (MELD) score. Upon admission to the surgery room, the patient is monitored using the bispectral index, electrocardiogram, pulse oximetry, noninvasive blood pressure, and central temperature. A peripheral venous line is inserted on which anesthetic induction takes place. All patients received balanced general anesthesia. Immediately after orotracheal intubation, a central venous catheter is inserted (preferably in the right internal jugular vein) and 2 arterial catheters (right femoral and right radial). During this time, the patient receives antibiotic prophylaxis and antifibrinolytic treatment with tranexamic acid (1 g at the beginning of surgery and 1 g at the start of the neohepatic phase). Anesthetic agents and catecholamines are administered as clinically indicated. The surgical incision begins only after following these steps. The surgical technique used is the standard one, with retrohepatic clamping of the inferior vena cava. In no patient the piggyback technique or the modified piggyback technique was used. Hemodynamic optimization was achieved by monitoring parameters obtained by the transpulmonary thermodilution method using the VolumeView/EV1000™ system, Edwards Lifesciences. Due to the lack of the necessary infrastructure, the management of coagulopathy in the past was based on standard coagulation tests, but currently we use ROTEM A5 Liver Algorithm.^[19]^ Throughout the surgical intervention, we try to keep the hemoglobin above 8 g/dL, the temperature above 35 °C, the pH and calcium within normal limits for all patients. Induction of immunosuppression is performed using Solumedrol and Basiliximab and for maintenance we use Mycophenolate mofetil and Tacrolimus. In the postoperative period, all patients are treated in a special isolated room dedicated to their care. For the prophylaxis of deep vein thrombosis, all patients have lower limb intermittent pneumatic compression and, depending on the risk of bleeding they receive or not low molecular weight heparin.

All patients were monitored postoperatively by a multidisciplinary team involving anesthesiology and intensive care, transplant surgery, and gastroenterology, with other specialist consultations provided when clinically indicated. After hospital discharge, follow-up was primarily coordinated by the gastroenterology team (initially at 1 week, then monthly or as often as necessary) while surgical or anesthetic input was provided when required. Each patient had a dedicated institutional transplant record in which all clinical assessments, laboratory tests, imaging studies, complications, and outcomes were systematically documented. Mortality status at 30 days and 1 year was confirmed through institutional medical records.

### 2.4. Collected data

For all patients, we recorded:

- Demographics: age, gender.
- Anthropometric data: weight, height and body mass index.
- Indication for liver transplantation: hepatocarcinoma or liver cirrhosis.
- Etiology of liver disease: toxic, viral, others (nonalcoholic steatohepatitis-NASH, Wilson, autoimmune, Budd-Chiari).
- Associated comorbidities: neurological (history of stroke), respiratory (chronic obstructive pulmonary disease, pleural effusions), preexisting cardiovascular conditions (arterial hypertension, pulmonary hypertension, valvopathies, chronic heart failure), chronic kidney disease, diabetes.
- MELD score.
- Data about the surgical intervention: duration (time, in minutes, from orotracheal intubation to transfer of the patient to the intensive care unit), intraoperative blood loss (measured by the cell-saver, in the aspirator or estimating the losses from the surgical fields and gauzes), the transfusion requirement (the volume of blood and blood product received by the patient measured in milliliters), the warm ischemia time (the time elapsed between the removal of the graft from the preservation solution and the completion of the vascular anastomoses) and the cold ischemia time (the interval between “cross- clamping” and completion of cavo-cava and porto-portal anastomoses with their declamping).
- Bleeding in the first 24 hours was calculated as the sum of intraoperative blood loss + the amount of blood estimated to be lost postoperatively until 24 hours have been reached from the start of the surgical intervention (monitoring surgical drains and dynamic hematocrit).

Primary outcome was evaluated by analyzing mortality at 30 days and at 1 year and secondary outcome were medical and surgical complications.

Medical complications included:

- Major neurological complications: altered neurological status, seizures, cerebrovascular complications, and central pontine myelinolysis^[20]^.
- Noninfectious respiratory complications: pleural effusion, pulmonary edema, atelectasis, acute respiratory distress syndrome^[21]^.
- Oro-tracheal reintubation.
- Cardiovascular complications: arrhythmia, heart failure, myocardial infarction, thromboembolism.^[22]^
- Acute kidney injury – according to the KDIGO criteria^[23]^.
- The need for Continuous Renal Replacement Therapy (CRRT).
- Infections occurring within 1-year posttransplant, identified based on CDC/NHSN surveillance criteria.^[24]^ Diagnosis was established in the presence of clinical signs (fever, chills, cough, dyspnea); microbiological confirmation from relevant biological specimens; radiological findings. Infections included in the analysis were pneumonia, bloodstream infections, urinary tract infections, surgical site infections, and intra-abdominal infections. Infection was distinguished from colonization based on the presence of a host immune response.

Surgical complications included^[25]^:

- Postoperative bleeding.
- Thrombosis of large vessels.
- Biliary complications.
- Surgical reintervention.

### 2.5. Statistical analysis

Statistical analysis was performed using the Jamovi ver. 2.4 (2023). Continuous variables were presented as mean ± standard deviation if they had normal distribution and median if they had abnormal distribution. The categorical variables are presented as absolute value and percentage (%). We calculated the CBV for each patient and compared this value with the estimated blood loss during the first 24 hours after the start of liver transplantation using the Student *t* test for normal distributed data, whereas for those with non-normal distribution, the Mann–Whitney test was used. The *χ*^2^-test was used for categorical data. Univariate was carried out on a number of recorded variables, and odds ratio (OR) with 95% confidence interval (CI) was calculated. We considered *P* < .05 as statistically significant.

## 3. Results

### 3.1. Patients characteristics

The study group comprised 53 patients with a mean age of 48.3 ± 9.4 years. Most of the patients were males (34, 64.1%). All patients had liver cirrhosis and 10 patients (18.8%) associated intra-Milan hepatocellular carcinoma. Viral hepatitis caused by B + D infection was the main etiology of liver cirrhosis in our cohort, followed by chronic alcoholic consumption and virus C infection. Cardiovascular comorbidities (23 patients, 43.4%), respiratory comorbidities (12 patients, 22.6%) and diabetes (11 patients, 20.7%) predominated, but a significant number were also with neurological comorbidities (3 patients, 5.6% with history of stroke) and renal comorbidities (3 patients, 5.6% with chronic kidney disease). Regarding the comparison between the 2 subgroups, with MH (26 patients, 49.1%) or without MH (27 patients, 50.9%), there were no statistically significant differences regarding demographic data, patient comorbidities, etiology of liver cirrhosis or MELD score. The preoperative baseline characteristics of all patients and the comparative analysis between those with MH and those without MH can be seen in Table 1. The only parameters identified as significantly lower in patients with MH compared to those without MH were preoperative BMI (24.3 vs 26.6 kg/m^2^, *P* = .028) and thrombocytopenia (55.5 vs 75 × 10^3^/µL, *P* = .04).

**Table 1:** Preoperative baseline characteristics of patients included in the study.

Variable	All patients N = 53	With MH N = 26	Without MH N = 27	P-value
Age, years, mean ± SD	48.3 ± 9.4	48.1 ± 9.9	48.6 (9.1)	.83*
Male gender, n (%)	34 (64.2)	16 (61.5)	18 (66.7)	.69***
Weight, kg, mean ± SD	76.2 ± 14.5	73.5 ± 13.2	78.7 ± 15.4	.20*
Height, m, mean ± SD	1.72 (0.09)	1.73 (0.09)	1.71 (0.09)	.94*
BMI, kg/m^2^, mean ± SD	25.5 (3.9)	24.3 (3.5)	26.6 (4.1)	.03*
Comorbidities				
Neurological, n (%)	3 (5.6%)	2 (7.7%)	1 (3.7%)	.56***
Respiratory, n (%)	12 (22.6%)	8 (30.8%)	4 (14.8%)	.29***
Cardiovascular, n (%)	23 (43.4)	9 (34.6%)	14 (51.9%)	.21***
CKD, n (%)	3 (5.6%)	2 (7.7%)	1 (3.7%)	.53***
DM, n (%)	11 (20.7%)	6 (23.1%)	5 (18.5%)	.68***
Hepatocarcinoma, n (%)	10 (18.9%)	4 (15.4%)	6 (22.2%)	.52***
Liver cirrhosis etiology				
Toxic, n (%)	15 (28.3%)	6 (23.1%)	9 (33.3%)	.41***
HCV, n (%)	10 (18.9%)	7 (26.9%)	3 (11.1%)	.14***
HVB + D, n (%)	19 (35.8%)	10 (38.5%)	9 (33.3%)	.69***
Other, n (%)	9 (17%)	3 (11.5%)	6 (22.2%)	.30***
Pretransplant status				
MELD score, mean ± SD	17.6 (6.2)	17.7 (5.2)	17.5 (7.2)	.90*
Hemoglobin (g/dL), mean ± SD	11.6 (1.7)	11.3 (1.8)	11.8 (1.6)	.27*
Platelets ×10^5^, median (IQR)	67 (56)	55.5 (53.7)	75 (72.7)	.04**
INR, median (IQR)	1.54 (0.48)	1.52 (0.38)	1.57 (0.69)	.92**
Creatinine (mg/dL), median (IQR)	0.76 (0.31)	0.79 (0.44)	0.76 (0.24)	.67**
Albumin (g/dL), mean ± SD	3.2 (0.67)	3.15 (0.61)	3.29 (0.75)	.57*

Categorical variables are presented as number, (percentage) and continuous variables as mean ± standard deviation (SD) if they had normal distribution and median if they had abnormal distribution.

BMI = body mass index, CKD = chronic kidney disease, DM = diabetes mellitus, HB + DV = hepatitis B + D virus, HCV = hepatitis C virus, INR = international normalized ratio, IQR = interquartile range, other = VHB, NASH, Wilson, autoimmune, Budd-Chiari.

* *t* Student tests.

** Mann–Whitney test.

*** χ^2^-test.

### 3.2. Data about surgery, massive hemorrhage, and transfusion requirements

The median blood loss following OLT was 5500 mL (4650 mL), and 26 patients (49.1%) had MH. Patients with MH had longer cold ischemia time than those without MH (257 vs 190 minutes, *P* = .003). Warm ischemia time was not statistically significantly different between the 2 subgroups. Patients with MH required more autologous (950 vs 500 mL, *P* < .001) and allogeneic blood (1500 vs 670 mL, *P* < .001) and more plasma transfusions (700 vs 460 mL, *P* = .04). The number of patients requiring platelet transfusions was significantly higher in the MH group (17 vs 1 pts, *P* < .001). The amount of fibrinogen concentrates or cryoprecipitate, as well as the number of patients who required such products, showed no statistical significance. Table 2 summarizes the data on the surgical intervention, bleeding, and transfusion requirements.

**Table 2:** Surgical intervention, bleeding, and transfusion requirements.

Variable	All patients N = 53	With MH N = 26	Without MH N = 27	*P*-value
EBL (mL), median (IQR)	5500 (4650)	8500 (8625)	4000 (2000)	<.001**
Surgery duration (min), median (IQR)	480 (100)	485 (138)	480 (100)	.20**
Cold ischemia time (min), median (IQR)	210 (119)	257 (115)	190 (55)	.003**
Warm ischemia time (min), mean ± SD	56.6 ± 12.6	57.9 ± 10.4	55.4 ± 14.8	.48*
Allogenic blood, volume (mL), median (IQR)	950 (895)	1500 (1000)	670 (333)	<.001**
Autologous blood, volume (mL), median (IQR)	670 (611)	950 (938)	500 (400)	<.001**
FFP, volume (mL), median (IQR)	600 (525)	700 (675)	460 (150)	.04**
FFP, n (%)	17 (32.1)	14 (53.8)	3 (11.1)	.001***
Platelets, n (%)	18 (33.9)	17 (65.4)	1 (3.7)	<.001***
Cryoprecipitate, n (%)	8 (15.1)	4 (15.4)	4 (14.8)	.95***
Fibrinogen concentrate, (g), median (IQR)	2 (1)	3 (2)	2 (1.5)	.33**
Fibrinogen concentrate, n (%)	43 (81.1)	22 (84.6)	21 (77.8)	.55***

EBL = estimated blood loss, FFP = fresh frozen plasm, IQR = interquartile range, SD = standard deviation.

* *t* Student tests.

** Mann–Whitney test.

*** χ^2^-test.

### 3.3. Outcome data

Complications developed in 43 (81.1%) of the patients. Of these, 42 (79.2%) had medical complications, and 20 (37.7%) had surgical complications. The most common medical complications encountered were acute kidney injury (AKI) in 20 patients (54.7%), with 9 (17%) requiring CRRT, infectious complications in 28 patients (52.8%), and noninfectious respiratory complications in 20 patients (37.7%). The most frequently encountered surgical complications were posttransplant bleeding in 13 patients (24.5%), thrombosis of large vessels in 10 patients (18.9%), and biliary complications in 8 patients (15.1%). Reoperation was required in 12 patients (22.6%). In our cohort 30 days mortality was 9.4%, and 1-year mortality was 17%. Patients who had massive hemorrhage had statistically significantly more frequent cardiovascular (38.5% vs 7.4%, *P* = .007) and infectious complications (69.2% vs 37%, *P* = .009) in the postoperative period. Posttransplant bleeding (38.5% vs 11.1%, *P* = .021) and the need for surgical reintervention (34.6% vs 11.1%, *P* = .04) was also encountered more frequently in patients who had MH. In the analyzed group, mortality did not depend on the presence or absence of MH on the day of surgery. Data related to the outcome and the calculated risk of developing postoperative complications are summarized in Tables 3 and 4.

**Table 3:** Patient’s outcome.

Variable	All patients N = 53	With MH N = 26	Without MH N = 27	*P*-value
Medical complications (any), n (%)	42 (79.2)	21 (80.8)	21 (77.7)	.79
Neurological complications, n (%)	17 (32.1)	8 (30.8)	9 (33.3)	.84
Noninfectious respiratory complications, n (%)	20 (37.7)	12 (46.1)	8 (29.6)	.21
Orotracheal reintubation, n (%)	21 (39.6)	12 (46.1)	9 (33.3)	.34
Cardiovascular complications, n (%)	12 (22.6)	10 (38.5)	2 (7.4)	.007
AKI, n (%)	29 (54.7)	16 (61.5)	13 (48.1)	.33
CRRT, n (%)	9 (17)	6 (23.1)	3 (11.1)	.28
Infectious complications, n (%)	28 (52.8)	18 (69.2)	10 (37)	.009
Surgical complications (any), n (%)	20 (37.7)	12 (46.1)	8 (29.6)	.21
Posttransplant bleeding, n (%)	13 (24.5)	10 (38.5)	3 (11.1)	.02
Thrombosis of large vessels, n (%)	10 (18.9)	5 (19.2)	5 (18.5)	.94
Biliary complications, n (%)	8 (15.1)	6 (23.1)	2 (7.4)	.11
Surgical reintervention, n (%)	12 (22.6)	9 (34.6)	3 (11.1)	.04
Any complications, n (%)	43 (81.1)	21 (80.8)	22 (81.5)	.95
Mortality at 30 days, n (%)	5 (9.4)	3 (11.5)	2 (7.4)	.61
Mortality at 1 year, n (%)	9 (17)	6 (23.1)	3 (11.1)	.25

AKI = acute kidney injury, CRRT = continuous renal replacement therapy.

**Table 4:** Risk of complications after massive hemorrhage during liver transplantation.

Variable	OR	CI 95%	*P*-value
Cardiovascular complications	5.19	1.256–21.469	.005
Infectious complications	2.07	1.149–3.753	.008
Posttransplant bleeding	3.64	1.072–11.180	.02
Surgical reintervention	3.11	0.947–10.246	.08
Any complications	3.11	0.690–14.060	.11
Mortality at 30 days	1.15	0.283–1.138	.61
Mortality at 1 year	2.07	0.579–7.447	.24

CI = confidence interval, OR = odds ratio.

## 4. Discussion

Numerous studies have established the link between intraoperative bleeding, massive transfusion, and the complicated outcome of the liver transplant patient in the postoperative period. However, there is limited information regarding the specific impact of MH on postoperative complications and short- and medium-term mortality in the OLT patient from a brain-dead donor. In our cohort, the incidence of MH was higher than typically reported by high-volume centers, reflecting the early phase of our transplant program and several local factors (advanced portal hypertension in referred patients, use of the classical surgical technique and limited early access to viscoelastic testing). These challenges are common in developing programs. As experience has grown within the anesthesia and surgical teams, bleeding has decreased significantly. We believe our findings highlight the importance of early bleeding control strategies and may serve as a useful reference for other emerging transplant centers.

Our study demonstrated that in 2 similar groups in terms of age, anthropometric data, comorbidities, preoperative paraclinical data, patients with MH had more frequent cardiovascular complications, infectious complications, tended to bleed postoperatively and may require surgical reintervention.

The advantage of OLT surgery is that it is a type of semi-elective surgery in which blood loss is anticipated. Currently, we are always preparing with a rapid and warm infusion system, an autotransfusion system and in stock of the hospital are enough blood products. Currently, death by exsanguination on the operating table of is a rare event, even nonexistent.^[18]^ In our study group, there was no death during surgery.

Even if the incidence of MH in liver transplant patients has decreased significantly in recent years, in our cohort it remains substantial and patients often required numerous blood products. Currently, there are experienced centers that report that approximately 70% to 80% of patients did not receive any blood product. Obtaining these results was possible using fluid restriction, liberal use of vasopressor medications, and avoidance fresh frozen plasma transfusion.^[26]^

Identifying the predictive risk factors for MH is essential to classify the patient on the risk scale and to be able to have proactive management. Our study identified body mass index and thrombocytopenia as risk factors, results similar to those of Chen, K-D et al, who, additionally identified also hemoglobin level as a predictor of MH in patients with OLT.^[27]^ Other preoperative risk factors associated with increased blood loss, identified in other studies, were the severity of liver disease, MELD score and previous surgery.^[28]^

MH during OLT can significantly impact postoperative outcomes, leading to various complications. Cardiovascular complications were significantly higher in patients with MH. To explain this, we have identified several causes. Hemodynamic optimization and maintaining an adequate cardiac output is more difficult if the blood losses are greater.^[12]^ Electrolytes imbalances (hypokalemia, hyperkaliemia, hypocalcemia, hypomagnesemia) during the liver transplant surgery are more significant as the blood loss is greater. Furthermore, the patient with end-stage liver disease is anemic, has changes in the structure of the myocardium that affect its performance and contributes to the environment for adverse cardiac events after transplantation.^[29]^

Patients experiencing MH during OLT have an increased risk of postoperative infections due to multiple interrelated factors. Hemorrhage management often requires large volumes of blood transfusion, which can lead to transfusion-related immunomodulation that is associated with nosocomial infections and impaired wound healing.^[30]^ Coagulopathy and hypothermia not only exacerbate bleeding but also contribute to endothelial injury, increasing the susceptibility to infections due to microbial translocation and impaired tissue repair. The need to use invasive devices for a long time contributes to the higher incidence of infections in this patient population.^[31]^

To improve the OLT patient outcome, the use of all known anesthetic measures to reduce bleeding is crucial. So currently, throughout the surgery, the major objectives are to avoid acidosis, hypothermia and hypocalcemia, maintain low portal pressure by correlating it with central venous pressure, adequate fluid therapy, advanced intra- and postoperative hemodynamic monitoring.^[32]^ To these is added the rational administration of blood products and drugs with a hemostatic visa using the viscoelastic tests instead of standard coagulation tests. Viscoelastic tests, in addition to the proven effect of decreasing the transfusion of erythrocyte mass, plasma and platelets, may also have the ability to reduce the number of thrombotic complications.^[19]^ Furthermore, when platelet concentrates or fresh frozen plasma are administered during surgery with the aim of improving the standard coagulation tests, it increases portal pressure and can promote bleeding.^[16,17]^ The relationship between bleeding volume and transfusion requirements in our cohort reflects institutional practices, including the routine use of intraoperative cell salvage with delayed autologous reinfusion, controlled hemodilution with crystalloids and colloids, and restrictive transfusion strategies guided by clinical and hemodynamic criteria.

Our study has several important limitations. It is a retrospective study carried out in a center with limited experience in the management of this type of patients. The study group is small, which may have limited the statistical power of the analysis. Consequently, the findings should be interpreted with appropriate caution and confirmed by larger, multicenter studies. Coagulopathy management was performed using viscoelastic tests only in 12 (22.6%) patients. The length of stay in intensive care could not be used as a criterion because, particularly in our institution, the patient stays in the same unit until discharge and his management is permanently handled by the intensive care department in collaboration with surgery and gastroenterology.

## 5. Conclusion

Massive hemorrhage on the day of surgery worsens the orthotopic liver transplant patient from a brain-dead donor outcome, especially in patients with low BMI. Patients have more cardiovascular complications, infectious complications and surgical reintervention is required more frequently. To improve the outcome of this patient, the application of all known methods for reducing intraoperative bleeding is mandatory.

## Author contributions

**Conceptualization:** Mihaela Blaj, Adi-Ionut Ciumanghel, Ana-Maria Trofin.

**Data curation:** Iulian Buzincu, Irina Ciumanghel.

**Investigation:** Iulian Buzincu, Eliza Isabela Bărbuță.

**Methodology:** Adi-Ionut Ciumanghel, Ana-Maria Trofin.

**Software:** Irina Gîrleanu.

**Supervision:** Mihaela Blaj.

**Validation:** Cristian Dumitru Lupașcu.

**Visualization:** Adi-Ionut Ciumanghel, Irina Gîrleanu, Eliza Isabela Bărbuță.

**Writing – original draft:** Iulian Buzincu.

**Writing – review & editing:** Mihaela Blaj, Cristian Dumitru Lupașcu.
